# Study of the Noise Reduction Mechanism of Bionic Circular Arch Structures on the Blades of a High-Volumetric-Airflow Axial Flow Fan

**DOI:** 10.3390/biomimetics11020127

**Published:** 2026-02-10

**Authors:** Chun Shen, Shijie Hu, Dongjun Xu, Chengchun Zhang, Xiaowei Sun, Wen Cheng

**Affiliations:** 1 National Key Laboratory of Automotive Chassis Integration and Bionics, Jilin University, Changchun 130022, China; shench@jlu.edu.cn (C.S.);; 2 School of Automotive Engineering, Jilin University, Changchun 130022, China; 3 Key Laboratory of Engineering Bionics, Ministry of Education, Jilin University, Changchun 130022, China

**Keywords:** bionic, axial flow fan, noise reduction, aeroacoustics noise

## Abstract

While bionic sawtooth and wave structures effectively reduce aerodynamic noise on fixed airfoils, their efficacy on rotating fans is often limited. Inspired by the protrusion structures of dragonfly wings and the gentle circular arches of manta rays, this study proposes a novel bionic circular arch structure to suppress aeroacoustic noise in axial flow fans. Numerical simulations were validated against experimental data from a standard fan, showing a sound pressure level (SPL) deviation within 3 dB at the first blade passing frequency (BPF), confirming calculation accuracy. The results indicate that the bionic design reduces the total SPL by approximately 2.5 dB. Notably, in the human-sensitive frequency range of 1000–3000 Hz, noise reduction reaches up to 6.6 dB at the upstream monitoring point. Analysis of Root Mean Square (RMS) fluctuating pressure and Fourier transforms reveals that the bionic structure significantly mitigates noise source intensity at the blade tip. This design effectively reduces pressure disturbances at the first BPF and shrinks the high-intensity disturbance region of the boundary layer compared to the prototype.

## 1. Introduction

Axial fans with large-volume airflows are widely used in industries such as energy, power, metallurgy, and transportation. Operating noise is inevitably generated, among which aerodynamic noise is dominant [1]. The environmental noise pollution caused by fan noise has become a public health issue that cannot be ignored, and the requirements of noise design standards are becoming increasingly stringent.

Bionic structures can be used to reduce the noise of axial fans. Bionic structures such as the trailing edge serrations of owl wings and the wavy leading edges of humpback whale flippers have been extensively studied. Wang et al. [2] designed a bionic airfoil extracted from the cross-section of an owl wing, incorporating a serrated trailing edge, and compared with the smooth airfoil, the bionic airfoil achieved a 5.47 dB reduction in total sound pressure level. Cao et al. [3] investigated a bionic airfoil with sinusoidal serrations at the leading edge, where the wavelength of the serrations was 0.24 c (c is the chord length), and these sinusoidal serrations at the leading edge can reduce the resonance energy by more than 21 dB over a wide speed range. Chen et al. [4] designed a bionic airfoil with a wavy leading edge incorporating porous materials. The experimental results indicated that at a Mach number of 0.12, an angle of attack of 0°, and an amplitude-to-wavelength ratio of 2, the total sound pressure level of this bionic airfoil was reduced by 13.2 dB. Fan et al. [5] employed the LES (large eddy simulation) method to investigate the broadband noise suppression effect of the NACA 0012 airfoil with a wavy leading edge and found that when the incoming flow velocity was 40 m/s and the azimuth angle was 90°, the total sound pressure level was reduced by 9.5 dB. Zhou et al. [6] experimentally investigated the aeroacoustic performance of a flap model featuring flexible trailing-edge serrations. Their results demonstrated that flexible serrations effectively suppressed lateral flow intensity near the roots and optimized the flow structure, achieving an additional noise reduction of 2–3 dB compared to rigid serrations. Zhu et al. [7] demonstrated that a modified cylinder inspired by seal fur could effectively suppress regular vortex shedding and reduce tonal noise.

However, applying airfoil-based bionic designs to rotating fans often yields limited efficacy. For instance, Lei et al. [8] applied owl-inspired leading-edge serrations to fan blades but achieved only a modest 1.3 dB reduction in total SPL under constant static pressure. Sun et al. [9] designed a novel axial flow fan impeller with a bionic arc-shaped hub and bionic serrated leading blades, and compared with the prototype impeller, the optimized design increased air volume by 3.7% while simultaneously reducing the total sound pressure level by 2.3 dB. Tian et al. [10] proposed a bionic blade inspired by the butterfly’s outer edge profile, and their experiments indicated that the noise was reduced by 1.3 dB. Liang et al. [11] developed an axial fan blade with a forked trailing edge inspired by fish tail fins; while their optimized structure achieved a maximum energy saving of 7.5%, the aerodynamic noise was reduced by only 0.3–0.8 dB. Therefore, new bionic structures or bio-inspired design approaches still need to be introduced to further reduce the SPL level for actual rotating fans. Notably, it is found that the microstructural protrusions on the leading edge of dragonfly wings enable a reduction in aerodynamic noise. Ren et al. [12] discovered dark-colored patch-like protrusions at the wingtips along the leading edge of dragonfly wings, and these protrusions feature an elongated, curved polygonal outer contour which effectively dampens vibrations and reduces noise. Jiang et al. [13], inspired by the serrated structure on the leading edge of dragonfly wings, designed a bionic airfoil that reduced the total sound pressure level by 12.5 dB. Hu et al. [14] pointed out that the micro-protrusion structure of dragonfly wings can be applied to the design of propellers and impellers, thereby improving aerodynamic performance and reducing noise. Although studies on fixed airfoils, such as those by Wang et al. [2] and Fan et al. [5], have demonstrated significant noise reduction, these findings are not entirely transferable to the complex rotating flow fields of axial fans. Previous attempts to apply bionic structures to rotating fans, such as the works of Lei et al. [8], Sun et al. [9], Tian et al. [10], and Liang et al. [11], achieved only modest reductions in the total sound pressure level (approximately 0.3–2.3 dB). Furthermore, traditional modifications like trailing-edge serrations often require material removal or significant structural changes, which alters the blade profile and potentially impacts aerodynamic efficiency negatively [2,7].

To address these limitations, this study proposes a novel, additive bionic design applied to an actual large-volume axial flow ventilation fan for high-speed trains. Inspired by the “protrusion structure” at the root of dragonfly wings [15,16,17] and the “gentle circular arch structure” of manta rays [18,19], a circular arch structure is designed to enhance noise reduction efficiency without compromising aerodynamic performance. Based on the FW-H acoustic analogy established by Williams and Hawkings [20], numerical simulations were conducted to predict aeroacoustic performance. To validate this approach, noise experiments were performed on the prototype fan at its rated rotational speed, showing good agreement between experimental data and simulation results. Subsequently, the mechanism regarding the reduction in the first blade passing frequency (BPF) noise and the total sound pressure level was thoroughly investigated, providing technical support for the flow-induced noise reduction design of industrial axial flow fans.

## 2. Simulation Analysis

### 2.1. Axial Flow Fan Model and Computational Domain

**Figure 1:**
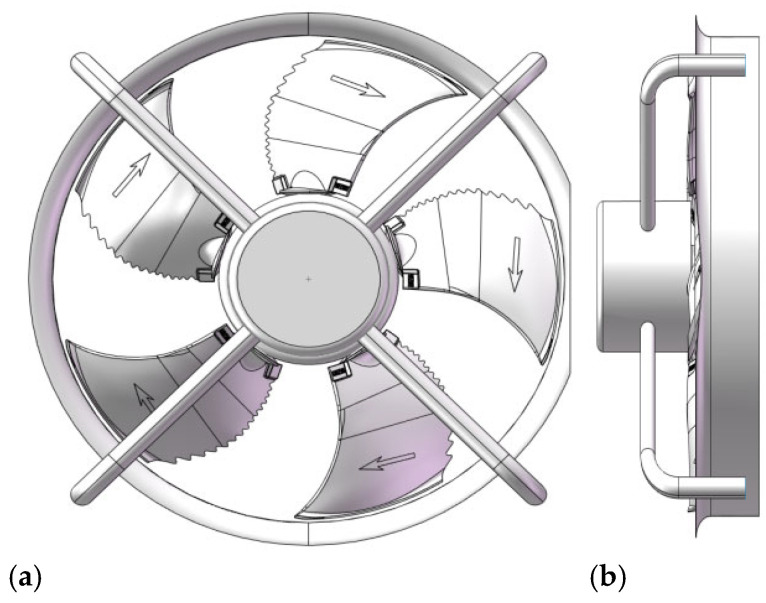
Simplified prototype axial flow fan model. (**a**) Front view at 90°. (**b**) Side view at 90°.

The model must first be simplified. Since the focus of this study is on analyzing the noise characteristics of the bionic-structured axial flow fan, all screw holes along the central axis of the fan are simplified, as they do not affect the comparison of noise levels. The simplified bionic axial flow fan model is shown in Figure 1.

**Figure 2:**
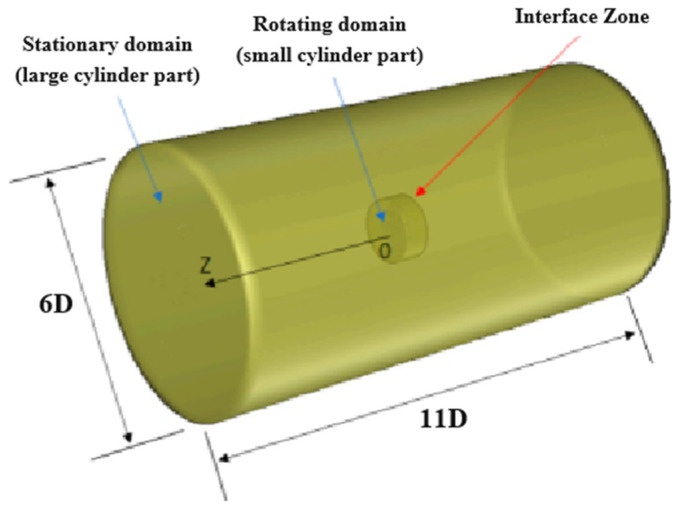
Instructions for the setting of the flow field domain.

The simulation computational domain of the axial flow fan consists of two regions: the rotating domain and the flow field domain. The rotating domain is positioned in the middle region of the flow field domain, with its length and width being 6 and 11 times the diameter of the fan, respectively. The computational domain is large enough to avoid the boundary effect. The specific schematic diagram is shown in Figure 2.

### 2.2. Introduction to the Design and Dimensions of the Arch Structure

**Figure 3:**
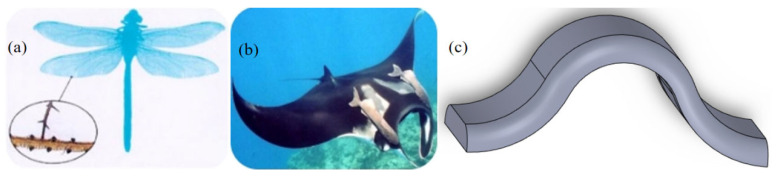
(**a**) The “bulge structure” at the trailing edge of the dragonfly’s wing. (**b**) The “gentle circular arch structure” of the manta ray’s circular arch. (**c**) The bionic circular arch structure.

As shown in Figure 3, by combining the “bulge structure” at the trailing edge of the dragonfly’s wings with the densely distributed characteristics of the leading edge of the owl’s wings, we arranged circular arch structures at equal intervals along the leading edge of the blade. The “gentle circular arch structure” of the bionic manta ray was also applied to the blade design, giving the entire blade a circular arch shape. This design ultimately creates multiple small circular arch holes on the pressure surface of the leading edge. During the blade’s rotation, the airflow is forced to pass through these circular arch holes, disrupting the average aerodynamic force on the blade surface, effectively reducing broadband noise and achieving overall noise reduction.

**Figure 4:**
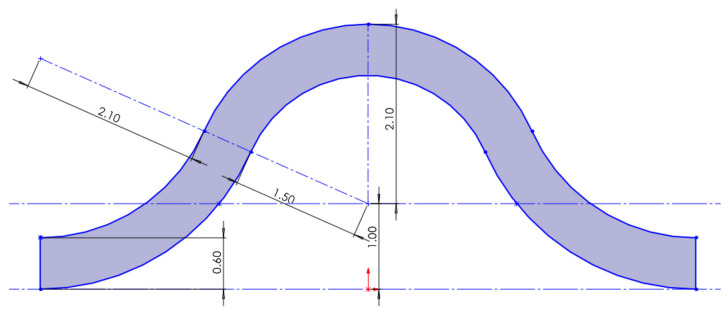
Sketch of the dimension design of the circular arch structure.

The dimensions of the bionic circular arch structure are shown in Figure 4. The inner diameter of the circular arch is 0.6 mm, the radius of the large arc is 2.1 mm, and the radius of the small arc is 1.5 mm. The vertical difference between the highest and lowest points is 3.1 mm, the boss extends by 3 mm, and the fillets on both sides have a radius of 0.5 mm.

**Figure 5:**
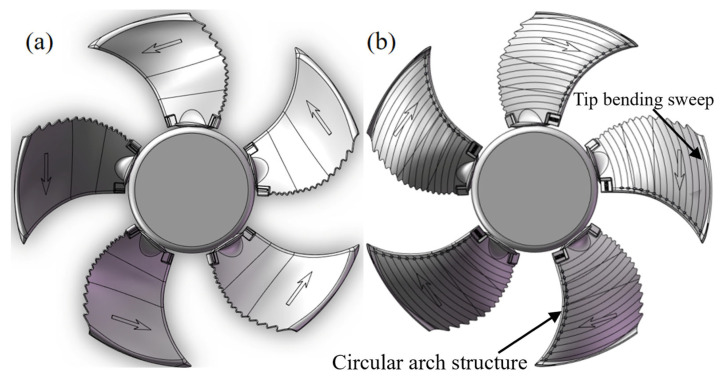
Simplified axial flow fan model with circular arch leading edge. (**a**) The pressure surface. (**b**) The suction surface.

**Figure 6:**
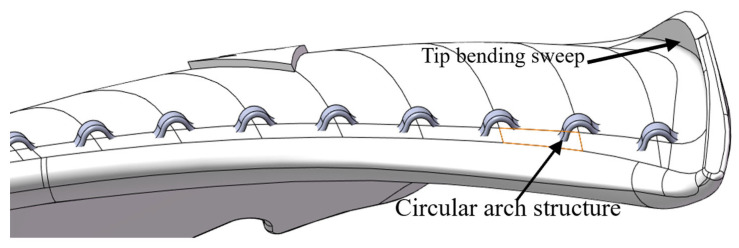
Detailed construction diagram of the blade with a circular arch leading edge structure.

Through simulation analysis of the RMS pulsating pressure and FFT surface load distribution at the first BPF peak on the blade surface in a basic fan, it is found that the primary noise sources of this axial flow fan are located in the leading edge and upper end areas of the blade suction surface, with the pressure effect at the leading edge of the suction surface being particularly significant. Therefore, the circular arch structure is arranged along the leading edge dividing line at 10 mm intervals, with the assembly position 3 mm away from the equivalent curve of the Z plane at the leading edge. A total of 14 individual blades are assembled. The design effect of the blade with the circular arch leading edge structure is shown in Figure 5, and the detailed structural diagram of the blade with the circular arch leading edge can be seen in Figure 6.

### 2.3. Mesh Generation of Axial Flow Fan

The hexahedral mesh offers good mesh quality and, due to its regular structure, simplifies post-processing. On the other hand, the polyhedral mesh is better suited for capturing the geometric features of curved surfaces and structures with holes under transient calculation conditions. It also provides greater stability in terms of the numerical solution’s stability and convergence. For the axial flow fan blades in this simulation, considering the advantages and disadvantages of each meshing method, STAR-CCM+ software (STAR-CCM+ 2210) is used to generate hexahedral meshes in the flow field domain, while polyhedral meshes are employed in the rotating domain.

In the mesh settings for the flow field domain, the target surface mesh size is 60 mm, with the minimum surface size also set to 60 mm. The thickness of the prism layer near the wall is 1 mm, and the number of prism layers is set to 10. The boundary layer growth rate is set to 1.2, resulting in a total prism layer thickness of 26 mm.

In the boundary condition settings, no prism layer is applied in the inlet and outlet regions.

**Figure 7:**
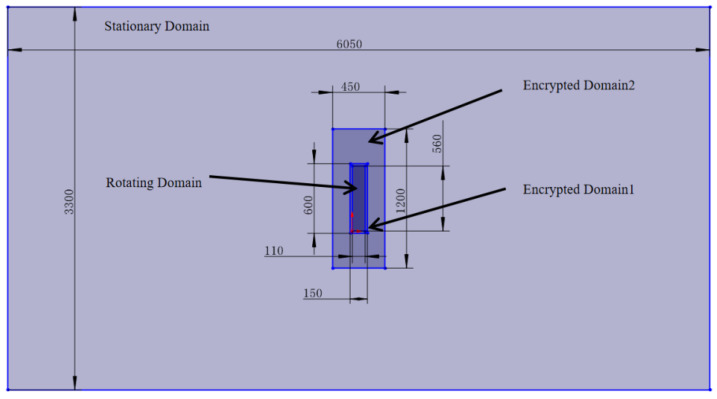
Schematic diagram of the dimensions of the encrypted region in the flow field domain.

In the encryption region settings, two cross-sections are established in the x-z plane and the x-y plane. The mesh in the flow field domain is refined with two-layer volume meshes. The target surface mesh size in the flow field domain of Encryption Region 1 is set to 10 mm, while in Encryption Region 2, the target surface mesh size is set to 2 mm. No prism layers are used in either case. This mesh division approach allows for more accurate results in the area close to the fan while simultaneously reducing the total number of meshes. The final mesh division of the flow field domain is shown in Figure 7.

**Figure 8:**
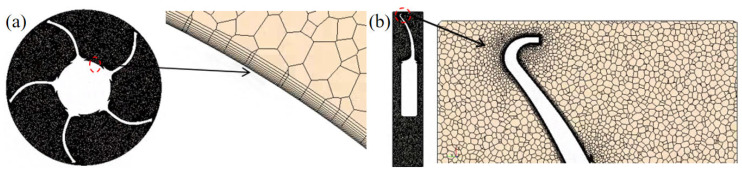
Schematic diagram of the mesh division in (**a**) the x-z plane and (**b**) the x-y plane of the rotating domain.

The mesh settings for the rotating domain and the fan surface are crucial, and as such, the mesh is defined with great detail. The target surface mesh size is set to 2 mm, with a minimum surface size of 2 mm. The prism layer near the wall has a thickness of 0.5 mm, consisting of 10 layers with a boundary layer growth rate of 1.2, resulting in a total prism layer thickness of 1.3 mm. No prism layers are applied in the inlet and outlet areas of the surface control. Two cross-sections are established in the x-z plane and the x-y plane. The mesh division details are shown in Figure 8.

**Figure 9:**
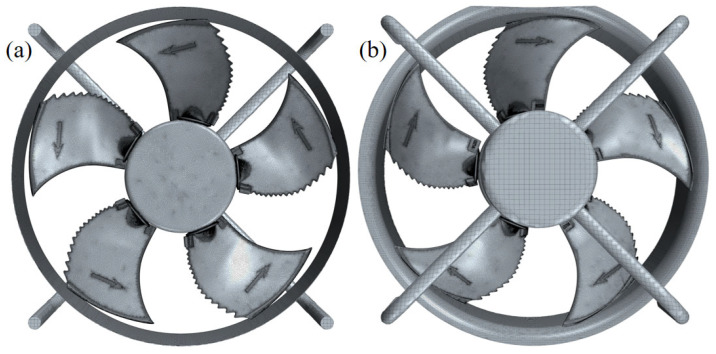
Schematic diagram of the planar mesh division of the prototype fan. (**a**) The pressure surface. (**b**) The suction surface.

For the fan surface, more detailed control is required. Based on the volume mesh settings of the rotating domain, surface control is applied to the fan blades and fan structure. The target surface mesh size is set to 0.2 mm, with a minimum surface size of 0.2 mm. The prism layer consists of 10 layers, with a near-wall thickness of 0.02 mm and a boundary layer growth rate of 1.2 resulting in a total prism layer thickness of 0.54 mm. For the bearing support at the center of the fan, the target surface size is set to 2 mm, with a minimum surface size of 2 mm. The prism layer settings for the bearing support are the same as those in the rotating domain. The mesh for the fan blades is shown in Figure 9.

The surface mesh counts for each region of the prototype and the circular arch leading edge are shown in Table 1 and Table 2.

### 2.4. Axial Flow Fan Flow Field Setting

#### 2.4.1. Flow Field Solution Setting

STAR-CCM+ software is used for simulating the flow field of the axial flow fan. The inlet condition is set as a stagnation inlet, meaning no physical quantities are input at the inlet. The outlet condition is defined as a pressure outlet boundary, with a relative gauge pressure of 0 Pa. All other boundaries are set as no-slip solid wall conditions, indicating that the surfaces in contact with the fluid are solid and there is no relative motion between them and the fluid. The rotating region is specified as a continuous fluid region, representing the rotating section inside the fan. To ensure the transfer of physical information between different regions, an in-place interface is set at the boundary between the inlet flow field domain and the rotating domain. The flow field calculation of the axial flow fan begins with a steady-state calculation to accelerate the convergence of the unsteady-state calculation and ensure the stability of the results.

In unsteady calculation settings, the Spalart–Allmaras DES (detached eddy simulation) model is used for unsteady flow calculation. The Spalart–Allmaras detached eddy model integrates the standard Spalart–Allmaras RANS model for boundary layers with a large eddy simulation (LES) approach in unsteady flow separation regions. To ensure numerical accuracy, a segregated solver was employed with second-order upwind spatial and second-order implicit temporal discretization schemes. The convergence criterion for continuity and momentum residuals was set to 10^−5^.

#### 2.4.2. Analysis of Flow Field Mesh Independence

First, a mesh independence analysis is performed. The inlet boundary condition is changed from a stagnation inlet to a velocity inlet, with the incoming flow velocity set to 1 m/s. The effect of mesh accuracy on the calculation results is assessed by comparing the pressure difference between inlet and outlet using a high-performance computing server equipped with AMD EPYC processors (AMD EPYC 7662 CPU, 64×2 cores) (128 cores, 512 GB RAM). The total wall-clock time required for the simulation was recorded for each mesh configuration to evaluate the computational cost. The results are shown in Table 3. Based on the verification from the mesh independence analysis, the mesh count for the prototype is deemed most appropriate at 17.31 million. Similarly, the mesh count for the cylindrical leading edge is set to 17.53 million, which is also considered to be an optimal number.

### 2.5. Acoustic Field Solution Setting

#### 2.5.1. Theoretical Background and Assumptions

Fundamentally, aerodynamic noise originates from unsteady flow structures in the near field, manifesting as hydrodynamic pressure fluctuations or “pseudo-sound” on the blade surfaces. Unlike acoustic waves, these near-field fluctuations do not propagate distinctively but decay rapidly with distance. According to the Ffowcs Williams and Hawkings (FW-H) acoustic analogy, these unsteady surface pressure loads act as dipole sources, serving as the primary mechanism for converting near-field hydrodynamic energy into propagating far-field acoustic waves at low Mach numbers. Therefore, the Root Mean Square (RMS) of surface pressure is analyzed in this study to identify the noise source intensity.

To predict far-field noise using the FW-H equation, three key assumptions are adopted in this simulation. First, acoustic decoupling is applied, neglecting the feedback of sound on the flow field. Second, considering the low-speed nature of the fan, the flow is characterized by a low Mach number, where dipole sources dominate the acoustic field; according to Lighthill’s scaling law, quadrupole terms (M^8^) are negligible compared to dipoles (M^6^), justifying the omission of volume integrals. Finally, free-field propagation is assumed, matching the anechoic experimental conditions.

#### 2.5.2. Acoustic Calculation Setup

After the unsteady calculation converges, the periodic pressure fluctuation on the rotor blades is selected as the sound source data for aeroacoustic calculations. Based on the flow conditions, the physical time step for the acoustic calculation must be at least one order of magnitude smaller than the characteristic time scale of the BPF. The rotational speed of the axial flow fan studied in this paper is set to 1415 rpm. The time-domain solution for the acoustic field information is obtained by solving the FW-H equation [20], and the frequency-domain solution of the acoustic field signal is derived through a Fourier transform. Through calculation, it is determined that the blade passing period is 0.05 s. The frequency range considered in this study is 100–20 kHz. Therefore, the noise calculation sampling time step is set to 5 × 10^−5^ s. The sound pressure level at the far-field noise monitoring point is subjected to a Fourier transform. The noise calculation time is 0.4 s, with 10 iterations per time step. The collection time starts at 0.25 s and ends at 0.4 s, with a total of 80,000 steps calculated.

**Figure 10:**
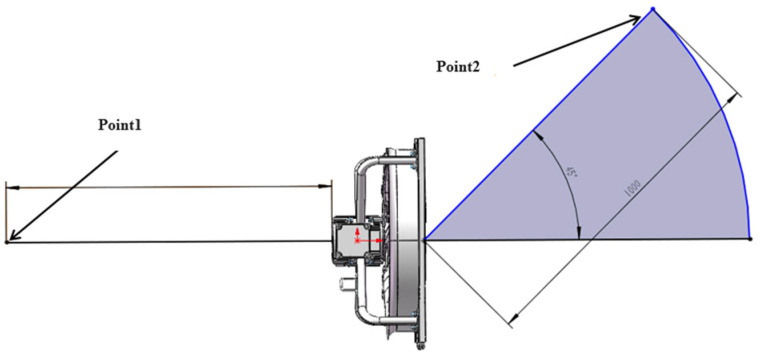
Distribution of noise monitoring points at upstream point 1 and at downstream point 2 of the prototype axial flow fan.

As shown in Figure 10, in accordance with national standard settings, two noise monitoring points are established. One is located 1 m from the inlet of the axial flow fan, and the other is positioned 1 m away at an upward angle of 45° from the air outlet of the axial flow fan.

## 3. Experimental Verification and Post-Processing Analysis

### 3.1. Experimental Model

**Figure 11:**
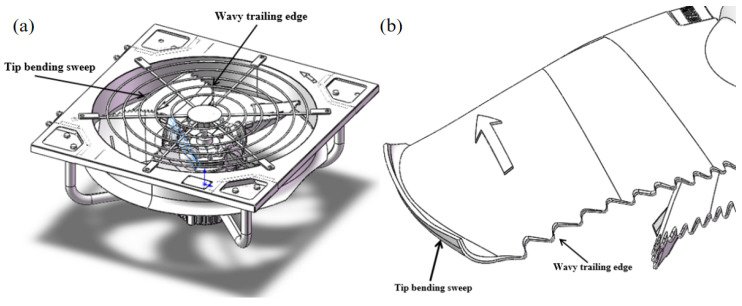
Schematic diagram of (**a**) the overall prototype axial flow fan and (**b**) the blade structure.

The trailing edge of the blade is designed to be wavy, which can reduce the turbulent noise generated at the trailing edge of the blade during the rotation of the blade [21,22,23]. The upper end of the blade adopts a sweep-back design. The application of the sweep-back can change the distribution of the static pressure gradient of the turbomachine and suppress the development of the secondary flow and the accumulation of low-energy fluid on the end wall, so as to achieve the purpose of improving the aerodynamic performance [24,25,26]. The model of the axial flow fan is designed as shown in Figure 11, and the specific parameters of the fan are shown in Table 4.

### 3.2. Measurement Conditions

#### 3.2.1. Measurement Environment

The measurement environment complies with the provisions of Chinese Standard GB/T 2888-2008 [27]. The experiment was conducted in the anechoic chamber of the China Aerodynamics Research and Development Center.

#### 3.2.2. The Arrangement of the Experimental Sound Source and the Position of the Microphone

**Figure 12:**
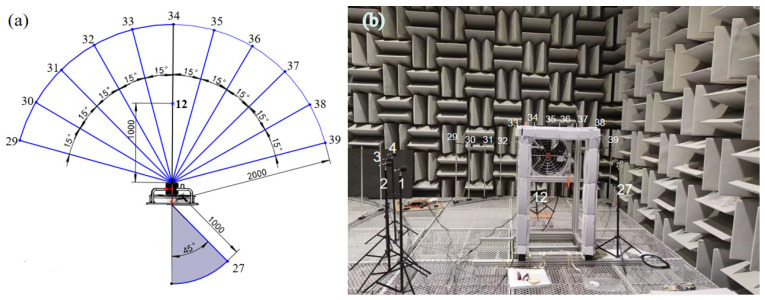
Schematic diagram of (**a**) the experimental microphone arrangement and (**b**) the corresponding layout of the experimental groups.

According to the “Measurement method for the noise of fans and roots blowers”, since the diameter of this axial flow fan is less than 1 m, the standard measurement distance is set to 1 m. Noise radiation is measured at upstream point 1 and downstream point 2, following the standard measurement distance of 1 m. Under the rated rotational speed of 1415 rpm, seven repeated experiments were conducted, with each experiment having an acquisition time of 30 s. Based on the national standard test requirements, the final microphone arrangement for the experiment and the actual setup of the anechoic chamber are illustrated in Figure 12. To verify the accuracy of the simulation, the upstream point 1 and the downstream point 2 are mainly benchmarked against experimental point 12 and point 27.

### 3.3. Results and Analysis of the Prototype Simulation and Experimental Acoustic Field Simulation

The sound pressure level (SPL), which quantifies sound intensity relative to a reference pressure, is defined as:

(1) $$SPL=20\log_{10}\left(\frac{p}{p_{ref}}\right)$$

where p is the measured sound pressure and $p_{ref}$ (20 μPa) is the reference pressure, representing the sound pressure level at the threshold of human hearing.

**Figure 13:**
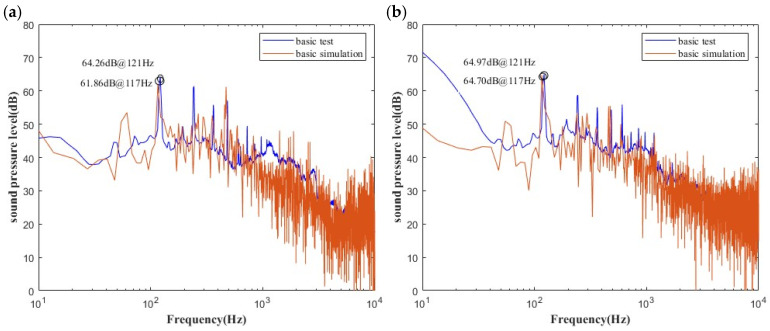
Schematic diagram of the comparison of power spectrum transformations at (**a**) point 1 and (**b**) downstream point 2 between simulation and test data.

Numerical simulations were carried out, with a time step of 5 × 10^−5^ s and a total of 80,000 iterations. FWH noise data collection commenced after 26,500 steps. A comparison between the simulation and experimental results is presented in Figure 13. The peak value of the sound pressure level at the inlet monitor point differs by 2.42 dB, while that at the air outlet differs by 0.27 dB, with the error remaining within 3 dB. Since the simulation omits minor structural details, such as surface screw holes and mounting brackets, the results are deemed reliable within the acceptable margin of error; that is, the numerical method meets the required calculation accuracy.

### 3.4. Results and Analysis of the Acoustic Field Simulation of the Bionic Fan

**Figure 14:**
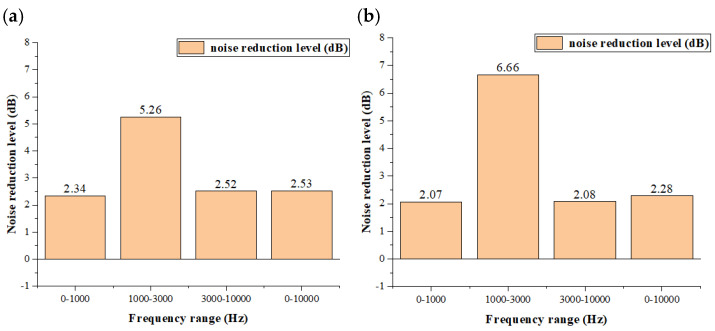
Diagram of the noise reduction amount of bionic fan with the circular arch structure in various frequency bands. (**a**) At upstream point 1. (**b**) At downstream point 2.

Table 5 and Figure 14 illustrate the noise reduction at upstream (point 1) and downstream (point 2) locations. The bionic fan exhibits significant noise suppression across multiple frequency bands compared to the prototype. Notably, in the frequency range of 1000–3000 Hz, the noise reduction is particularly significant, reaching 5.26 dB at the inlet and 6.66 dB at the outlet. Given that this frequency band coincides with the peak sensitivity of human hearing (highly correlated with A-weighted sound levels), the significant reduction in this range effectively lowers the perceived annoyance. Consequently, the bionic design mitigates the subjective noise impact more effectively than the reduction in overall broadband SPL alone would suggest.

**Figure 15:**
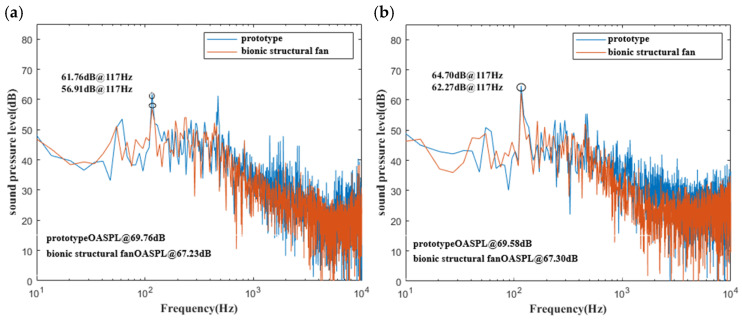
The comparison of sound pressure level of power spectrum transformations (**a**) at upstream point 1 and (**b**) at downstream point 2 between the prototype and the bionic fan.

A comparison between the SPL of power spectral density for the bionic and prototype fans is shown in Figure 15. The total sound pressure level is reduced by approximately 2.5 dB. Additionally, the SPL at the first BPF is reduced by 4.85 dB and 1.83 dB at upstream monitoring point 1 and downstream point 2, respectively, and it is indicated that the circular arch structure enable to reduce noise at the frequency corresponding to first BPF. Furthermore, the red spectrum curve is significantly lower than the blue spectrum within the 1000–3000 Hz range, and it coincides with the conclusion that there is a significant amount of noise reduction in the frequency range of 1000–3000 Hz as shown in Figure 15.

### 3.5. Analysis of the Noise Reduction Mechanism

#### 3.5.1. Comparative Analysis of Bionic Configurations and Parameter Sensitivity

**Figure 16:**
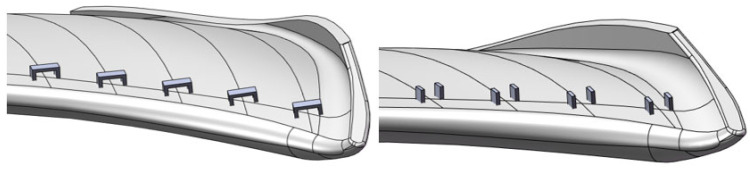
The control models with straight frame and straight column configurations.

To rigorously verify the unique noise reduction contribution of the “circular arch” morphology, two control models were designed as shown in Figure 16: the straight frame and the straight column.

As detailed in Table 6, all three bionic structures exhibited significant noise suppression in the sensitive 1000–3000 Hz band, with reductions reaching approximately 5–6 dB at both the inlet and outlet. However, regarding the total sound pressure level, the performance of the control models diminished significantly downstream. The straight column and straight frame achieved a reduction of only 0.52 dB at the outlet. In contrast, the circular arch maintained a superior performance, reducing the total SPL by 2.53 dB at the inlet and 2.28 dB at the outlet. This comparison confirms that while leading-edge protrusions facilitate high-frequency suppression, the streamlined circular arch morphology is essential for preventing secondary flow noise, thereby ensuring consistent broadband reduction.

**Figure 17:**
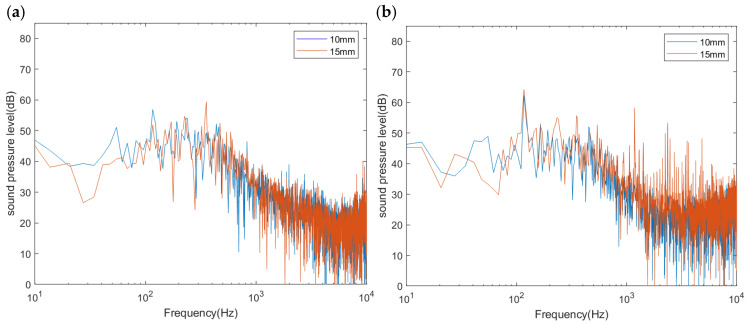
Comparison of sound pressure level spectra between 10 mm and 15 mm spacing configurations at (**a**) upstream point 1 and (**b**) downstream point 2.

Based on the superior performance of the circular arch morphology, the influence of the distribution spacing was further investigated to verify the rationality of the design parameters. A comparative simulation was conducted with the spacing interval increased from 10 mm to 15 mm, while maintaining other geometric dimensions unchanged. The comparison of the total sound pressure level (SPL) is presented in Table 7, and the specific spectral characteristics are illustrated in Figure 17.

Table 7 presents the quantitative comparison. The results indicate that increasing the spacing to 15 mm compromises the noise suppression effect. Specifically, while the 15 mm design reduces upstream noise, its downstream (point 2) total SPL rises to 70.41 dB, which exceeds the prototype’s level (69.58 dB). In contrast, the 10 mm configuration achieves consistent reductions at both monitoring points. Therefore, the 10 mm spacing is confirmed as the optimal parameter for this study.

#### 3.5.2. Analysis of Flow Field and Noise Source Mechanisms

**Figure 18:**
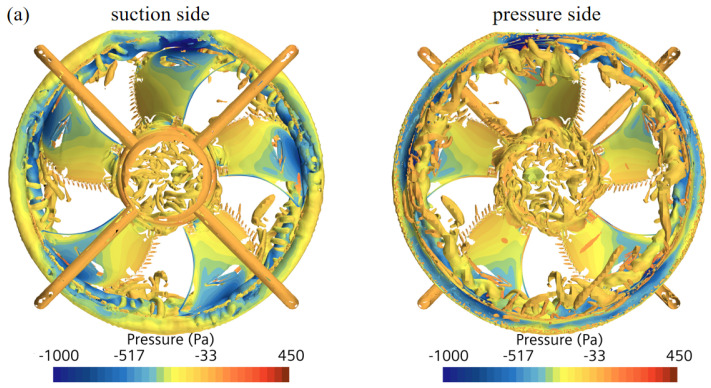

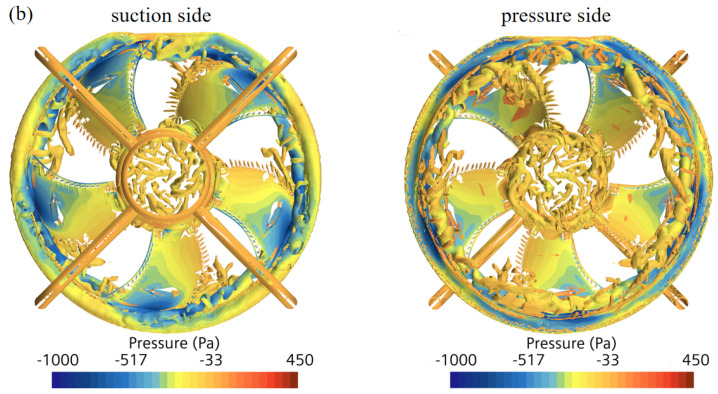
Iso-value of the Q-criterion (300,000). (**a**) The prototype. (**b**) The circular arch.

Figure 18 displays the Q-criterion contours for the prototype and the circular arch fan. Densely concentrated vortex cores are observed in the tip clearance region. While the macro-scale vortex structures appear similar, the significant noise reduction implies that the bionic design functions by altering local surface pressure fluctuations rather than suppressing global flow separation.

Fluctuating pressure, representing the deviation from the mean value, is expressed as:

(2) $$p^{\prime }=p-\bar{p}$$

where $p^{\prime }$ is the fluctuating pressure, p is the instantaneous pressure, and $\bar{p}$ is the time-averaged pressure.

RMS pressure serves as a reliable indicator of fluctuation intensity. For n data points ${p_{1}}^{\prime }$, ${p_{2}}^{\prime }$, …, ${p_{n}}^{\prime }$, the RMS fluctuating pressure is defined as:

(3) $${p_{RMS}}^{\prime }=\sqrt{\frac{1}{n}\sum_{i=1}^{n}{p_{i}}^{\prime 2}}$$

where ${p_{RMS}}^{\prime }$ is the RMS fluctuating pressure, ${p_{i}}^{\prime }$ is the pressure data sample, and n is the number of samples.

**Figure 19:**
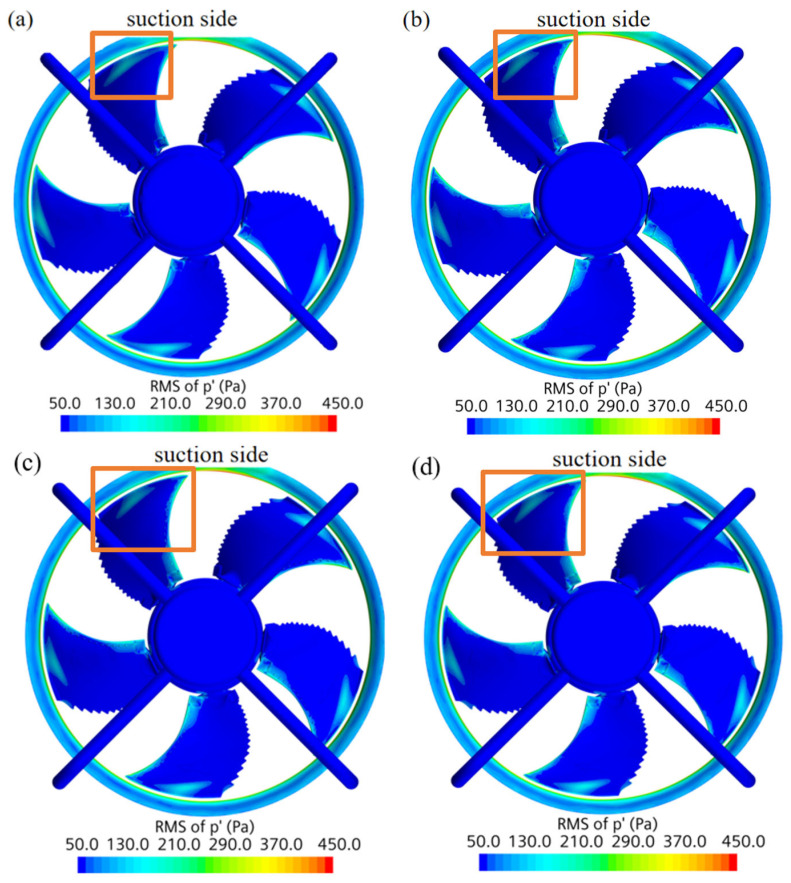
Contour of the RMS of the fluctuating pressure. (**a**) Prototype. (**b**) Circular arch. (**c**) Straight frame. (**d**) Straight column.

Figure 19 illustrates the surface RMS pressure distribution. Since high-intensity noise sources are predominantly located at the leading edge and tip region of the suction surface, the analysis focuses on this area.

Comparing the four models, while the control models (straight frame and straight column) reduce source intensity, the circular arch structure most effectively shrinks the high-amplitude noise region at the blade tip. Its visibly smaller high-intensity region confirms superior capability in suppressing surface pressure fluctuations.

To investigate noise source characteristics at the first BPF, the fast Fourier transform (FFT) was applied. The Fourier transform, converting a signal from the time domain to the frequency domain, is defined as:

(4) $$F(\omega )=\int_{-\infty }^{\infty }f(t)e^{-i\omega t}dt$$

where $e^{-i\omega t}$ is the exponential term in Euler’s formula, i is the imaginary unit, ω is the frequency, and t is time. This formula represents the integral of the product of the function f(t) and the exponential function $e^{-i\omega t}$ over the entire time domain.

**Figure 20:**
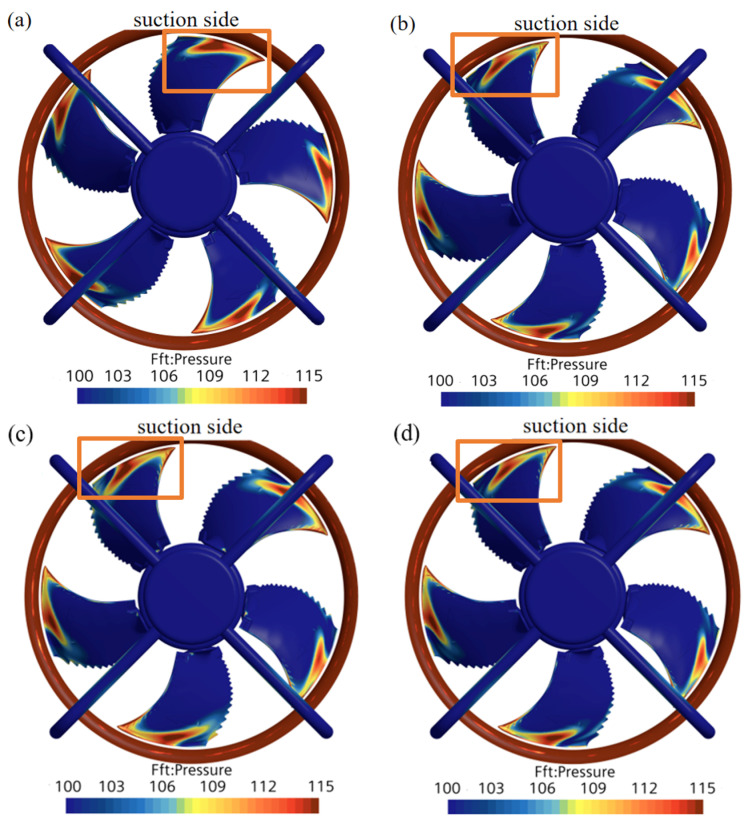
Contour of the FFT surface load distribution at the first BPF. (**a**) Prototype. (**b**) Circular arch. (**c**) Straight frame. (**d**) Straight column.

Figure 20 presents the SPL contours at the first BPF. The high-load area at the suction side tip is significantly smaller for the circular arch model compared to the control models. The straight frame model retains a large high-load area, indicating that its non-streamlined upper connecting structure likely induces secondary disturbances, partially counteracting noise reduction. In contrast, the circular arch’s streamlined morphology conforms to the flow, minimizing secondary aeroacoustic sources. The minimized high-SPL area at the first BPF corresponds to the maximum reduction in source amplitude. Consequently, the upstream far-field SPL of the bionic fan decreases by 4.85 dB at the first BPF, outperforming the straight frame (2.95 dB) and straight column (1.02 dB) models.

### 3.6. Analysis of the Rotational Flow Field of the Prototype and Analysis of the Noise Reduction Mechanism

**Figure 21:**
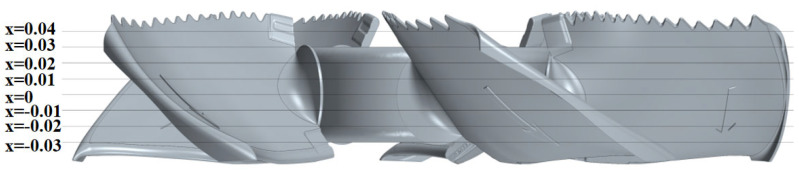
Schematic diagram of the distribution positions of various cross-sections of the prototype fan.

To study the distribution of noise sources in the spatial flow field, eight cross-sections are evenly distributed along the fan’s axial flow direction, i.e., from x = −0.03 to x = 0.04, in Figure 21. The middle cross-section of the fan is x = 0.

**Figure 22:**
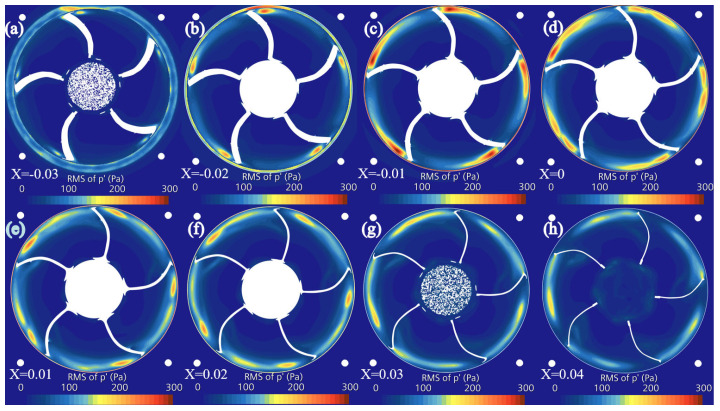
Contour of the RMS of the fluctuating pressure of various cross-sections of the prototype fan. (**a**) x = −0.03. (**b**) x = −0.02. (**c**) x = −0.01. (**d**) x = 0. (**e**) x = 0.01. (**f**) x = 0.02. (**g**) x = 0.03. (**h**) x = 0.04.

The RMS value of the fluctuating pressure effectively reflects the unsteady characteristics in fluid flow. By analyzing the RMS values of fluctuating pressure across each blade cross-section, the intensity of the system’s noise and vibration can be revealed. Higher RMS values typically correspond to larger noise and vibration amplitudes, thus indicating changes in the spatial distribution of noise sources. This analysis helps to verify and further explore previous research findings. The schematic diagram of the RMS values of fluctuating pressure for each cross-section is shown in Figure 22. As observed from the figure, the disturbance source at the center of the blade (x = 0) exhibits the highest values, with the high-value disturbance starting at the leading edge. In the sections (x = −0.01) and (x = 0), the disturbance source intensity in the tip trailing-edge flow field exhibits higher peak values and a larger influence range. Closer to the leading- and trailing-edge side sections, namely (x = −0.03) and (x = 0.04), the disturbance source intensity in the tip wake is lower.

**Figure 23:**
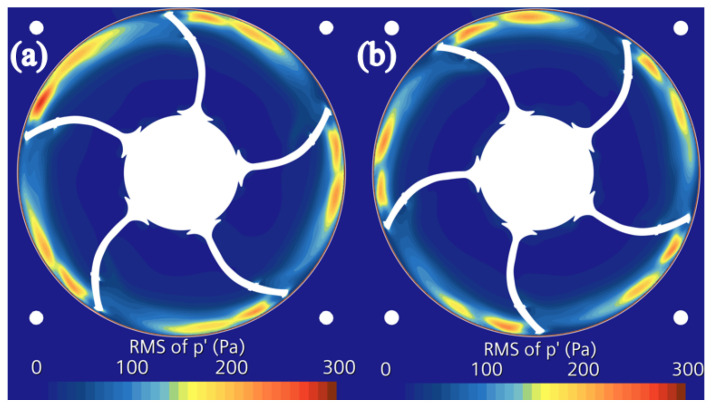
Contour of the RMS of the fluctuating pressure of various cross-sections of the fan at the fan center (x = 0). (**a**) The prototype fan. (**b**) The bionic fan.

According to the analysis of the prototype flow field, the magnitude of the disturbance source at the middle cross-section of the blade (x = 0) is the largest. Further, the RMS of the fluctuating pressure at this cross-section of the blades for both the prototype fan and the circular arch leading-edge fan is compared, as shown in Figure 23. It is clear that compared to the prototype fan, the high-value disturbance sources corresponding to the bionic fan with the circular arch leading edge structure on the blade are significantly reduced. Notably, the area of the high-value region for the bionic fan is relatively small, which confirms the superior noise reduction effect of the bionic circular arch on the total sound pressure level in the sound field.

## 4. Conclusions

This study initially validated the accuracy of the numerical simulation by comparing it with experimental data. Subsequently, a thorough analysis utilizing numerical simulation and post-processing was performed to reveal the underlying mechanism responsible for the reduction in the first BPF noise and the overall sound pressure level in the bionic-structure fan. Through experimental and simulation studies, it is found that:

1. A hybrid computational method combining DES and the FWH acoustic analogy is employed to predict the aerodynamic noise of the original fan prototype. Comparing the SPL values predicted by the numerical method with experimental data, a maximum difference of 2.42 dB was observed at upstream point 1 and 0.27 dB at downstream point 2. The difference in SPL between the numerical method and experiment at the first BPF is within 3 dB, indicating that the calculation accuracy of the numerical method meets the required standards for aeroacoustic analysis.
2. The primary noise sources on the surface of the axial flow fan are located at the leading edge of the suction surface of the blade and the tip of blade, and the high value of the RMS is particularly located at the middle of the blade tip. Additionally, noise sources on the pressure surface are concentrated near the blade tip at the trailing edge and the curved part of the blade tip.
3. According to the distribution of the noise sources, the bionic circular arch structures are designed and placed at the leading edge of the suction surface. The total sound pressure level is reduced by approximately 2.5 dB. Since the noise source is suppressed directly at the blade tip, this reduction propagates into the far field according to the acoustic inverse-square law. Consequently, the design effectively mitigates annoyance for observers at any distance. Furthermore, a significant noise reduction of 5 dB is achieved within the frequency band of 1000–3000 Hz, which is the most sensitive range for the human ear. Additionally, the SPL at the first BPF is reduced by 4.85 dB and 1.83 dB at upstream point 1 and downstream point 2, respectively. This indicates that the circular arch structure effectively mitigates noise at the first BPF.
4. Through the analysis of the RMS distribution, the high-amplitude noise sources at the blade tip of the bionic fan are notably diminished. Fourier transform analysis reveals that at the first BPF, there is a significant reduction in the SPL of the pressure disturbance at the tip of the bionic blade surface. Moreover, the results of the RMS distribution of the boundary layer pressure disturbance sources suggest that the high-intensity disturbance region close to the blade of the bionic fan is remarkably smaller than that of the prototype fan.

## Figures and Tables

**Table 1:** Mesh division of the simulation calculation domain of the prototype axial flow fan.

Region Name	Number of Meshes
Flow field domain	6.8 million
Rotating domain	10.51 million
Total number of volume meshes	17.31 million

**Table 2:** Mesh division of the simulation calculation domain of the bionic axial flow fan.

Region Name	Number of Meshes
Flow field domain	6.65 million
Rotating domain	10.88 million
Total number of volume meshes	17.53 million

**Table 3:** Analysis of mesh independence.

Total Number of Meshes	Number of Meshes in the Flow Field Domain	Number of Meshes in the Rotating Domain	Pressure Difference Between Inlet and Outlet	Total Calculation Time
13.94 million	6.79 million	9.14 million	2.14 pa	63 h
16.67 million	6.79 million	9.88 million	2.16 pa	75 h
17.31 million	6.79 million	10.52 million	2.20 pa	78 h
20.69 million	6.79 million	13.90 million	2.19 pa	93 h

**Table 4:** Specific parameter table of the fan.

Parameter	Size
Fan diameter	550 mm
Diameter of bearing during rotation of fan	196 mm
Angle of attack during rotation of blade	18°
Tip clearance	3 mm
Number of blades	5
Rotational speed	1415 rpm
Rated voltage/current of the motor	380 V/2.4 A

**Table 5:** Summary table of the noise reduction effects in various frequency spectrum ranges of the bionic fan.

Frequency (Hz)	The Prototype		The Bionic Fan		Comparison of Noise Reduction Amount	
	Point 1 (dB)	Point 2 (dB)	Point 1 (dB)	Point 2 (dB)	Point 1 (dB)	Point 2 (dB)
0–1000	69.19	68.96	66.85	66.89	2.34	2.07
1000–3000	59.00	58.2	53.73	51.54	5.26	6.66
3000–10,000	55.57	57.36	53.04	55.28	2.52	2.08
0–10,000	69.76	69.58	67.23	67.30	2.53	2.28

**Table 6:** Comparison of noise reduction effects in different frequency bands among three bionic structures.

Frequency (Hz)	Straight Column Reduction		Straight Frame Reduction		Circular Arch Reduction	
	Point 1 (dB)	Point 2 (dB)	Point 1 (dB)	Point 2 (dB)	Point 1 (dB)	Point 2 (dB)
0–1000	1.02	0.18	1.10	0.19	2.34	2.07
1000–3000	5.95	6.19	6.26	6.17	5.26	6.66
3000–10,000	2.47	2.14	2.95	1.80	2.52	2.08
0–10,000	1.32	0.52	1.42	0.52	2.53	2.28

**Table 7:** Comparison of sound pressure level (SPL) between different spacing configurations.

Frequency (Hz)	The Prototype		Circular Arch (10 mm)		Circular Arch (15 mm)	
	Point 1 (dB)	Point 2 (dB)	Point 1 (dB)	Point 2 (dB)	Point 1 (dB)	Point 2 (dB)
0–1000	69.19	68.96	66.85	66.89	67.51	69.17
1000–3000	59.00	58.2	53.73	51.54	53.35	62.79
3000–10,000	55.57	57.36	53.04	55.28	52.8	59.23
0–10,000	69.76	69.58	67.23	67.30	67.81	70.41

## Data Availability

The data presented in this study are available on request from the corresponding author.
